# A novel programme to evaluate and communicate 10-year risk of CHD reduces predicted risk and improves patients' modifiable risk factor profile

**DOI:** 10.1111/j.1742-1241.2008.01872.x

**Published:** 2008-10

**Authors:** J S Benner, L Erhardt, M Flammer, R A Moller, N Rajicic, K Changela, C Yunis, S B Cherry, Z Gaciong, E S Johnson, M C J M Sturkenboom, J García-Puig, X Girerd

**Affiliations:** 1 IMS Health, Inc., Falls Church, VA, USA and the Center for Clinical Epidemiology & Biostatistics, University of Pennsylvania School of MedicinePhiladelphia, PA, USA (at the time this study was conducted); 2 Lund UniversityMalmö, Sweden; 3 Pfizer IncNew York, NY, USA; 4 Medical University of WarsawWarsaw, Poland; 5 Graunt & Co.LLC, Seattle, WA, USA; 6 Department of Medical Informatics and Epidemiology & Biostatistics, Erasmus University Medical CenterRotterdam, The Netherlands; 7 Vascular Risk Unit, Division of Internal Medicine, Hospital Universitario La PazMadrid, Spain; 8 Division of Endocrinology, Hôpital Pitié Salpêtrière, Assistance Publique-Hôpitaux de ParisParis, France

## Abstract

**Aims:**

We assessed whether a novel programme to evaluate/communicate predicted coronary heart disease (CHD) risk could lower patients' predicted Framingham CHD risk vs. usual care.

**Methods:**

The Risk Evaluation and Communication Health Outcomes and Utilization Trial was a prospective, controlled, cluster-randomised trial in nine European countries, among patients at moderate cardiovascular risk. Following baseline assessments, physicians in the intervention group calculated patients' predicted CHD risk and were instructed to advise patients according to a risk evaluation/communication programme. Usual care physicians did not calculate patients' risk and provided usual care only. The primary end-point was Framingham 10-year CHD risk at 6 months with intervention vs. usual care.

**Results:**

Of 1103 patients across 100 sites, 524 patients receiving intervention, and 461 receiving usual care, were analysed for efficacy. After 6 months, mean predicted risks were 12.5% with intervention, and 13.7% with usual care [odds ratio = 0.896; p = 0.001, adjusted for risk at baseline (17.2% intervention; 16.9% usual care) and other covariates]. The proportion of patients achieving both blood pressure and low-density lipoprotein cholesterol targets was significantly higher with intervention (25.4%) than usual care (14.1%; p < 0.001), and 29.3% of smokers in the intervention group quit smoking vs. 21.4% of those receiving usual care (p = 0.04).

**Conclusions:**

A physician-implemented CHD risk evaluation/communication programme improved patients' modifiable risk factor profile, and lowered predicted CHD risk compared with usual care. By combining this strategy with more intensive treatment to reduce residual modifiable risk, we believe that substantial improvements in cardiovascular disease prevention could be achieved in clinical practice.

**What's known**

Guidelines for CVD prevention generally recommend the assessment and management of overall cardiovascular risk. However, recommendations are not consistently implemented by many doctors, and patients frequently do not meet therapeutic targets for modifiable risk factors such as blood pressure and lipids. Novel strategies are urgently required to help improve reductions in modifiable risk factors achieved by patients in clinical practice, and thus lower their overall risk of CHD.

**What's new**

A novel risk evaluation/communication programme incorporating the concept of global predicted risk was associated with greater reductions in 10-year predicted risk, higher levels of blood pressure and lipid goal attainment, and an increase in smoking cessation, compared with usual care. This risk evaluation/communication strategy could mediate improvements in CVD prevention in clinical practice, although to achieve substantial benefits it needs to be combined with more intensive medical treatment for multiple risk factors.

## Introduction

Coronary heart disease (CHD) is the most prevalent form of cardiovascular disease (CVD) and the most common cause of death worldwide (1). Hypertension, dyslipidaemia and cigarette smoking are the three modifiable risk factors most strongly and independently associated with CHD (2,3). Pharmacologic and lifestyle interventions targeting these risk factors have been shown in large clinical trials and meta-analyses to reduce the risk of CHD death (4–8). However, despite the proven benefits of such interventions, improvements in modifiable risk factors remain suboptimal in clinical practice (9–14). Potential reasons for this treatment gap are lack of physician implementation of guidelines and poor patient adherence to recommended drug therapies or lifestyle modifications (14–18). In particular, despite advances in therapies in recent years, improvements in lifestyle factors among patients at risk for CVD have been minimal (14). Novel strategies for CVD prevention are urgently required to help motivate patients and physicians towards improving the reductions in modifiable risk factors achieved in clinical practice, and thus lower patients' overall risk of CHD.

Evidence-based guidelines, such as those from the Fourth Joint Task Force of European and Other Societies on Cardiovascular Disease Prevention in Clinical Practice (19) and the National Cholesterol Education Program (NCEP) Adult Treatment Panel (ATP) III (20), have emphasised the importance of considering overall cardiovascular risk in CVD management. Frequently, usual care in clinical practice involves the treatment of individual risk factors to target levels, however, a more global approach is needed to effectively lower patients' overall risk (18). Global risk equations, including the Framingham (21), Prospective Cardiovascular Munster (22) and Systematic COronary Risk Evaluation (23) models, can estimate overall CVD/CHD risk based on a patient's age, sex, blood pressure, cholesterol, smoking status and other cardiovascular risk factors. While some previous studies have investigated the benefits of assessing and communicating patients' predicted CVD/CHD risk for improving risk factor management, these interventions have had limited benefits (24–26).

Recently, the large EuroAction trial evaluated the efficacy of a nurse-led, multidimensional approach that aimed to educate and support coronary patients and those at risk for CVD to comply with guideline-recommended treatments and lifestyle changes. Compared with patients receiving usual care, patients receiving this intervention showed significant improvements in cardiovascular risk factors and compliance with lifestyle changes (27–29). Several other studies evaluating strategies designed to educate or motivate physicians and patients have reported positive changes in patient behaviour and efficacy outcomes as a result of similar interventions (30–37).

We developed a novel CHD risk evaluation and communication programme that aimed to incorporate the concept of global predicted risk into a comprehensive intervention strategy designed both to facilitate clinical decision making by physicians and to educate and motivate patients to reduce their global cardiovascular risk. This intervention programme utilises the Framingham risk calculation for the assessment of 10-year CHD risk. Although the Framingham model is not intended to assess treatment effects, we believe that this can provide a useful tool to communicate a patient's global risk, and that relative changes in predicted Framingham 10-year risk following intervention can provide an indication of the potential achievable benefits if improvements are maintained over the long term. The Risk Evaluation And Communication Health Outcomes and Utilization Trial (REACH OUT) was therefore conducted to evaluate the clinical utility of our CHD risk evaluation/communication programme for lowering CHD risk as measured by Framingham risk equations, in comparison with usual care, in a multi-country setting (38).

## Methods

### Study design

The REACH OUT study was a 6-month, parallel group, prospective, controlled, cluster-randomised, multinational trial conducted between September 2005 and November 2006. The study design has been described in more detail in a previous publication (38). Briefly, study sites in nine European countries (Czech Republic, Denmark, France, Germany, Greece, The Netherlands, Poland, Spain and Sweden) were randomised in a 1 : 1 ratio to deliver either usual care following screening or a CHD risk evaluation and communication programme.

A cluster-randomised design was selected in order that physicians were not required to administer both usual care and the intervention, as physician education during the intervention programme may have influenced their approach to usual care. Cluster randomisation was performed by randomly selecting pairs of study sites within each block of 10 sites using a computer-based algorithm, and randomly assigning them to the intervention or usual care arm. At each site physicians screened patients from an alphabetical list of potential study participants, taken from the physicians’ files, to identify 100 patients who met initial criteria. Using a random permutation of 100 numbers, patients were then sequentially screened and consented until 10–15 patients had been enrolled per site. All study sites were required to adhere to Good Clinical Practice/International Conference on Harmonization guidelines, and the study protocol was reviewed and approved by the relevant ethics committee in each participating country.

### Inclusion/exclusion criteria

Patients had to be 45–64 years of age with a history of hypertension, systolic blood pressure ≥ 140 mmHg (or ≥ 130 mmHg for patients with renal disease) (39,40), and a 10-year risk of myocardial infarction (MI) or death because of CHD of ≥ 10% as predicted by the Framingham equation (41). Exclusion criteria included a history of CHD or diabetes mellitus, or a fasting plasma glucose > 6.9 mmol/l (124 mg/dl) at screening. Physicians were required to be certified General Practitioners or Internists who were not using CHD risk algorithms in their routine practice prior to the study.

### Programmes

In both the usual care and intervention groups, screening was conducted by recording patients' characteristics and risk factors using a small, portable, touch-screen computer [Touch Outcomes Collector (TOC); ASSIST Technologies, Scottsdale, AZ]. The TOC analysed the patient's risk factor profile and calculated the Framingham risk, which was the basis for inclusion in the study.

**Figure 1:**
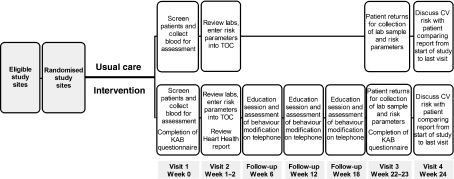
Study flow diagram. KAB, Knowledge, Attitudes and Behaviour; TOC, Touch Outcomes Collector; CV, cardiovascular

In the usual care group, physicians were provided with the study protocol, which included an explanation of the purpose of the trial, and received training on the use of the TOC. Predicted Framingham 10-year risk of CHD was calculated but was not communicated to either the physician or patient until the final visit, when the patient's risk at baseline and at month 6 was reviewed with the patient by the physician (although for ethical reasons, laboratory results used to assess risk were available to the physician during the study period), and physicians were instructed locally to provide usual care only during the study period (Figure 1).

In the intervention group, physicians received training in risk assessment and communication as part of an investigator meeting prior to enrolling patients (usual care physicians were not invited to participate). At weeks 1–2, physicians were informed of patients' predicted CHD risk and were instructed to advise the patient according to a CHD risk evaluation and communication programme (Figure 1). This was designed to inform patients of their predicted absolute and potentially modifiable 10-year risk of CHD (excluding the impact of age and sex on predicted risk), and to educate them about modifiable risk factors and their control through behavioural changes and drug therapy. As part of the intervention, patients received a Heart Health Report (38) (Figure S1) generated by the TOC and based on the patient's risk factor profile, which was reviewed with the physician at the same visit (Figure 1). The report illustrated modifiable risk with a bar chart comparing the patient's predicted absolute risk with that of a non-smoker of the same age and sex with ‘normal’ untreated blood pressure (defined in this study as systolic blood pressure = 139 mmHg) and cholesterol levels [total cholesterol = 4.9 mmol/l (189 mg/dl); high-density lipoprotein cholesterol (HDL-C) = 1.0 mmol/l (40 mg/dl) for males or 1.2 mmol/l (46 mg/dl) for females] (38).

The intervention also involved three follow-up phone calls by a physician or study nurse at weeks 6, 12 and 18, and patients completed a ‘Knowledge, Attitudes and Behaviour’ (KAB) questionnaire at week 0 and week 22–23 (designed specifically for the purposes of this trial) (Figure 1). Responses to 3–5 multiple-choice questions within each domain of the KAB questionnaire were summed, with possible scores in the range of 0–5 for knowledge, 5–25 for attitudes and 3–15 for behaviour; higher scores indicating more positive responses. A fourth domain was included with multiple-choice questions designed to assess patient satisfaction with the intervention and physician compliance with the protocol. The Heart Health Report and KAB questionnaire have been described in more detail previously (38).

In both the usual care and intervention groups, all medications (including cardiovascular medications) were prescribed at the discretion of the physician.

### Efficacy measures

#### Primary efficacy measure

The primary efficacy measure was the Framingham 10-year predicted risk of MI or CHD death at month 6 in the intervention group vs. the usual care group. Calculation of 10-year predicted risk of CHD was based on the Framingham model employed in the NCEP ATP III risk assessment tool (41); variables included age, sex, systolic blood pressure, total cholesterol, HDL-C, antihypertensive treatment status and smoking status.

#### Secondary efficacy measures

To assess the intervention's ability to reduce excess risk attributable to modifiable risk factors, the difference in ‘modifiable risk’ was compared between the intervention and usual care groups. Modifiable risk was defined as a patient's predicted 10-year risk in excess of the risk for a ‘normal’ individual, defined as a non-smoker of the same age and sex, not receiving antihypertensive treatment, and with normal cholesterol and blood pressure (as defined above for the TOC). This was calculated as [(r_1_ −r_2_)/r_2_] × 100%, where r_1_ is the risk for the patient, and r_2_ is the risk for the normal individual. Therefore, a patient with twice the predicted risk of a normal individual would have a modifiable risk of 100%. As the parameters used to define r_2_ were values below which risk could be further reduced with intensive treatment (42–50), our definition of r_2_ and thus our definition of modifiable risk were conservative.

Additional secondary measures included changes in blood pressure and lipids, attainment of blood pressure (< 140/90 mmHg) and low-density lipoprotein cholesterol [LDL-C; < 3.4 mmol/l (130 mg/dl)] goals (20,39,40), weight loss, and changes in smoking status. Adverse events (AEs) were also monitored.

### Statistical analyses

The study was designed to enrol a total of 110 sites each with 11 evaluable patients, to provide at least 80% power to detect a 10% relative difference in the primary end-point of Framingham 10-year risk at month 6 at a 5% significance level. Standard deviation of the primary end-point was assumed to be 0.35 (on the natural logarithm scale).

Given the cluster-randomised design, the intra-class correlation (ICC) of patients from the same site was considered, where an ICC of 0.05 was used in the calculation. This sample size was estimated using two Stata (version 8.2; College Station, TX, USA) programs: sampsi and sampclus, and confirmed using statistical simulation methods.

The primary end-point of the Framingham 10-year predicted risk of CHD at month 6 was evaluated using a mixed effects model (SAS® MIXED procedure; SAS Institute, Cary, NC, USA). As the distribution of the Framingham risk score is often highly skewed, the natural logarithm of the primary efficacy end-point was used as the response variable, with the study group, country, gender, age and a logarithm of the 10-year predicted Framingham risk at baseline as covariate terms in the model.

The primary analysis was the calculation of a two-sided p-value, and the 95% confidence interval, for the difference between least squares mean values for the intervention and usual care groups at month 6.

## Results

### Patient population

**Figure 2:**
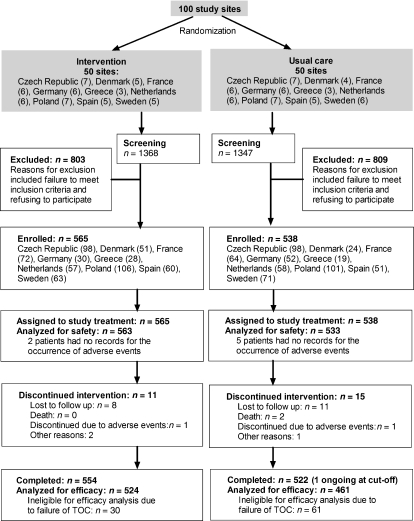
Patient flow. AEs, adverse events; TOC, Touch Outcomes Collector

Overall, 101 study sites were randomised to deliver usual care or the risk evaluation and communication programme. Of these, one site in the intervention arm was excluded because of not returning the appropriate local approval documentation. From the 100 sites included in the analysis (50 in each group), 1103 patients were recruited; 1076 completed the study and 985 were eligible for inclusion in the primary efficacy analysis (461 allocated to usual care; 524 patients allocated to intervention) (Figure 2). Of the 118 patients excluded from the efficacy analysis, for 91 patients this was due to failure of the TOC to capture the data correctly; 26 patients discontinued from the study and one patient was ongoing at cut-off (Figure 2). The estimated sample size of 1210 patients was not met; however, as the observed dropout rate was lower than predicted, the study remained adequately powered.

Among the overall study population, mean age was 57 years and approximately 14% of patients were female. On average, patients were overweight (mean body mass index was 29 kg/m^2^), and over half were smokers. While 18% had a history of dyslipidaemia according to patients' medical records, 51% were retrospectively classified with dyslipidaemia based on their lipids and medications at screening. Only 3% had a prior history of non-CHD cardiac disorders (Table 1).

**Table 1:** Baseline characteristics

Characteristics	Usual care (*n* = 538)	Intervention (*n* = 565)	Total (*n* = 1103)
Female, *n* (%)	83 (15.4)	71 (12.6)	154 (14.0)
Age (years), mean (SD)	56.9 (4.9)	56.8 (5.1)	56.8 (5.1)
White*, *n* (%)	534 (99.3)	525 (92.9)	1059 (96.0)
Weight (kg), mean (SD)	87.8 (15.6)	87.8 (15.0)	87.8 (15.3)
Height (cm), mean (SD)	173.2 (8.6)	173.3 (8.5)	173.2 (8.5)
BMI (kg/m^2^), mean (SD)	29.2 (4.6)	29.2 (4.5)	29.2 (4.5)
SBP† (mmHg), mean (SD)	158.6 (14.0)	157.0 (14.0)	157.8 (14.0)
DBP† (mmHg), mean (SD)	94.0 (9.7)	93.3 (8.4)	93.6 (9.1)
Total cholesterol† (mmol/l), mean (SD)	5.9 (1.0)	5.9 (1.0)	5.9 (1.0)
LDL-C† (mmol/l), mean (SD)	3.9 (1.0)	3.9 (0.9)	3.9 (0.9)
HDL-C† (mmol/l), mean (SD)	1.4 (0.4)	1.4 (0.4)	1.4 (0.4)
Triglycerides† (mmol/l), mean (SD)	2.1 (1.3)	2.1 (1.2)	2.1 (1.3)
History of dyslipidaemia, *n* (%)	93 (17.3)	101 (17.9)	194 (17.6)
Dyslipidaemia according to lipids/medication use, *n* (%)	267 (49.6)	300 (53.1)	567 (51.4)
Smoker†, *n* (%)	257 (55.7)	273 (52.1)	530 (53.8)
History of non-CHD cardiac disorders‡, *n* (%)	19 (3.5)	15 (2.7)	34 (3.1)
10-year predicted risk of CHD*, mean (SE)	16.9 (0.26)	17.2 (0.25)	17.1 (0.17)

* p < 0.01.

† Data are for the efficacy analysis population only (*n* = 461 usual care; 524 intervention).

‡ The most common cardiac disorder in both groups was arrhythmia (1.6% intervention and 3.0% usual care). To convert mmol/l to mg/dl divide by 0.02586 for total cholesterol, LDL-C and HDL-C values, and by 0.01129 for triglycerides. SD, standard deviation; SBP, systolic blood pressure; DBP, diastolic blood pressure; LDL-C, low-density lipoprotein cholesterol; HDL-C, high-density lipoprotein cholesterol; CHD, coronary heart disease.

For patients included in the efficacy analysis, at the beginning of the study, 82.4% of usual care patients were receiving cardiovascular medications; 80.9% were receiving antihypertensives and 23.9% were on serum lipid-reducing medications. Among intervention patients, 81.5%, 77.9% and 27.1% were taking cardiovascular medications, antihypertensives and serum lipid-reducing agents, respectively (Table 3). More than half of patients in both groups were receiving agents acting on the renin–angiotensin system at screening.

**Table 3:** Percentage of patients receiving medications at screening and at the end of the study

Patients receiving medications, *n* (%)	Usual care (*N* = 461)			Intervention (*N* = 524)		
	Screening	Final visit	% Change	Screening	Final visit	% Change
Any cardiovascular medications	82.4	93.3	13.2	81.5	89.1	9.4
Any antihypertensive	80.9	91.1	12.6	77.9	84.7	8.8
ACE inhibitors	29.3	33.0	12.6	22.0	27.3	24.3
ACE inhibitor combinations	5.0	6.5	30.4	4.8	5.9	24.0
ARBs	14.3	17.4	21.2	14.1	16.0	13.5
ARB combinations	8.5	10.6	25.6	10.9	13.4	22.8
Beta-blocking agents	37.1	41.2	11.1	29.4	31.3	6.5
CCBs	22.1	27.3	23.5	23.9	28.8	20.8
Diuretics	28.0	32.5	16.3	15.3	20.2	32.5
Other antihypertensives	6.5	6.7	3.3	5.0	6.7	34.6
Serum lipid-reducing agents	23.9	38.8	62.7	27.1	42.0	54.9
Aspirin	4.1	5.2	26.3	8.2	10.9	32.6
Peripheral vasodilators	3.0	3.5	14.3	1.5	1.5	0
Anti-obesity medications	0	0	0	0.4	0.4	0
Others*	2.4	4.1	72.7	2.7	4.0	50.0

* Including antihaemorrhoidals for topical use, corticosteroids and flavonoids. ACE, angiotensin-converting enzyme; ARB, angiotensin receptor blocker; CCB, calcium channel blocker.

### Predicted 10-year risk of non-fatal MI or CHD death

After 6 months, the mean (SE) predicted 10-year risk of non-fatal MI or fatal CHD was reduced from baseline [16.9% (0.26) in the usual care group; 17.2% (0.25) in the intervention group], to 13.7% (0.27) and 12.5% (0.25) among usual care and intervention patients respectively (Table 2). This corresponds to a relative decrease in predicted risk of 18.2% in the usual care group, compared with 25.7% among patients receiving the intervention.

**Table 2:** Change in absolute and modifiable Framingham 10-year risk of CHD

	Usual care (*N* = 461)	Intervention (*N* = 524)
**Absolute Framingham risk**		
Baseline, mean (SE)	16.9 (0.26)	17.2 (0.25)
Month 6, mean (SE)	13.7 (0.27)	12.5 (0.25)
Adjusted† change in risk from baseline to month 6, LS mean (95% CI)**	−4.9§ (−5.5 to −4.3)	−6.3§ (−6.9 to −5.7)
**Modifiable Framingham risk (% above ‘normal’)‡**		
Baseline, mean (SE)	221 (15.7)	192 (12.4)
Month 6, mean (SE)	117 (9.3)	87 (7.8)
Adjusted† change in risk from baseline to month 6, LS mean (95% CI)*	−96§ (−115 to −78)	−115§ (−133 to −97)

* p < 0.05.

** p < 0.001, for intervention vs. usual care.

§ p < 0.001 vs. baseline.

† Data were adjusted for baseline risk and other covariates.

‡ Defined as the risk for a non-smoker of the same age, gender, not receiving antihypertensive treatment and with total cholesterol = 4.9 mmol/l (189 mg/dl), high-density lipoprotein cholesterol = 1.0 mmol/l (40 mg/dl) for males or 1.2 mmol/l (46 mg/dl) for females = 1.3 mmol/l (50 mg/dl), and systolic blood pressure = 139 mmHg (38). SE, standard error; LS, least square; CI, confidence interval.

Following adjustments for baseline risk and other covariates (including country, age, gender and baseline risk), predicted absolute 10-year risk of CHD at month 6 in the intervention group was 0.896 times that in the usual care group (95% CI: 0.84–0.96; p = 0.001) (Table 2). The mean change in predicted absolute risk for the intervention vs. usual care was −6.3 in the intervention group and −4.9 in the usual care group, corresponding to a between-group difference of −1.4 (95% CI: −2.1 to −0.8; p< 0.001; ICC = 0.033).

### Modifiable 10-year risk of non-fatal MI or CHD death

The changes in modifiable risk of CHD (i.e. the risk attributable to cardiovascular risk factors that can be altered by lifestyle changes and/or medication) in the usual care and intervention groups are given in Table 2. At baseline, patients in the usual care and intervention groups had a modifiable CHD risk of 221% and 192%, respectively (i.e. on average their predicted risk was approximately three times that of a non-smoker of the same age and sex with normal untreated blood pressure and lipids). After 6 months, the modifiable risk was substantially lowered in both groups but was still 117% in the usual care group, vs. 87% in the intervention group. After adjusting for baseline risk and other covariates, the mean absolute difference in the change in modifiable risk with the intervention vs. usual care was −18.5% (95% CI: −35.5 to −1.4; p = 0.034) (Table 2).

### Changes in modifiable risk factors and weight loss

**Figure 3:**
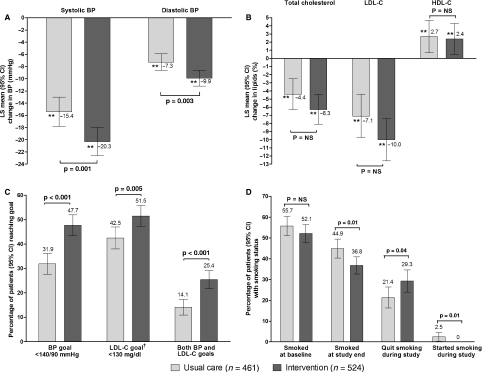
Changes in modifiable risk factors. (A) Change in blood pressure. (B) Change in lipids. (C) Attainment of blood pressure and LDL-C goals. (D) Change in smoking status. **p < 0.0001 vs. baseline. ^†^At baseline, 28.9% and 29.8% of patients in the usual care and intervention groups respectively, were at LDL-C goal; < 1% of patients were at blood pressure goal at baseline. BP, blood pressure; LS, least square; CI, confidence interval; TC, total cholesterol; LDL-C, low-density lipoprotein cholesterol; HDL-C, high-density lipoprotein cholesterol

Over the 6 month study period, mean blood pressure was reduced from 159/94 to 144/87 mmHg in the usual care group, and from 157/93 to 138/85 mmHg in the intervention group. After adjusting for baseline values and other covariates, blood pressure was reduced significantly more among patients receiving the intervention than among those receiving usual care (p< 0.01) (Figure 3A).

Baseline LDL-C was approximately 3.9 mmol/l (∼150 mg/dl) in both groups, which was reduced to 3.5 mmol/l (135 mg/dl) and 3.4 mmol/l (131 mg/dl) in the usual care and intervention groups respectively, after 6 months. After adjustments, a slightly greater decrease in LDL-C was observed in the intervention group than with usual care, although the difference between groups was not significant (p = 0.052) (Figure 3B). Similarly, total cholesterol was reduced from approximately 5.9 mmol/l (228 mg/dl) in both groups to 5.6 mmol/l (216 mg/dl) in the usual care group compared with 5.4 mmol/l (211 mg/dl) in the intervention group; the between-group difference for these reductions was not significant (p = 0.095) (Figure 3B). From baseline to month 6, there was a non-significant increase in triglycerides of 3.7% (95% CI: −2.8 to 10.3; p = 0.26) in the usual care group, and 3.2% (95% CI: −3.1 to 9.4; p = 0.32) in the intervention group (p = 0.88 between groups).

Overall, a significantly higher proportion of patients in the intervention group achieved blood pressure and LDL-C goals than in the usual care group (Figure 3C). Patients in the intervention group were 1.9 times (95% CI: 1.2 to 2.9; p = 0.003) as likely to attain their blood pressure goal; 1.6 times (95% CI: 1.2 to 2.2; p = 0.005) as likely to attain LDL-C goal and 2.1 times (95% CI: 1.3 to 3.3; p = 0.002) as likely to attain both goals as patients in the usual care group.

Within the intervention group, 97% of smokers were encouraged to stop smoking by the physician (6.3% of all patients were prescribed anti-smoking medications). Among these patients, 29.3% were reported to have quit smoking at the end of the study, compared with 21.4% of smokers in the usual care group (p = 0.04) (Figure 3D).

Across both treatment groups, reductions in predicted modifiable risk (Table 2) were driven predominantly by decreases in systolic blood pressure (accounting for 25.5% of the risk reduction), followed by changes in total cholesterol (21.6% of the risk reduction), and quitting smoking (15.7% of the risk reduction), while only 7.8% of the modifiable risk reduction was due to increases in HDL-C (percentages are based on analysis of variance components from a regression model; the difference from the sum of percentages to 100% is the remainder of the variability unexplained by the model).

Within the intervention group, after adjusting for covariates at baseline, patients achieved a mean decrease in weight of 1.14 kg (95% CI: 1.71 to 0.56), which although marginal, was statistically significantly greater than the mean change in weight among usual care patients [−0.35 kg (95% CI: −0.95 to 0.25); p= 0.015].

### Patient responses to KAB questionnaire

At month 6, among patients in the intervention group, mean scores increased by 7%, 5% and 12%, from baseline values of 4.4, 21.8 and 11.6 (p < 0.0001 from baseline for all) for the knowledge, attitudes, and behaviour domains of the questionnaire, respectively. Increased scores for attitudes correlated significantly (p = 0.014) with reductions in modifiable Framingham risk, while knowledge and behaviour scores showed no significant correlation with modifiable risk reduction. In addition, scores for attitudes and behaviour, but not knowledge, correlated significantly (p < 0.0001 and p = 0.0048) with decreases in blood pressure. None of the scores correlated with lipid changes. Over three-quarters of patients (77.5%) reported that their physician spent > 5 min explaining the Heart Health Report. When asked their favourite part of the intervention in domain 4 of the questionnaire, 31.9% of patients said the additional time with their doctor, 14.5% the personal report, 4.0% the follow-up calls and a further 30.9% enjoyed all three equally (16.0% did not answer this question and 2.7% responded ‘none of the above’).

### Change in medications from screening to the end of the study

Over the study period, cardiovascular medications were prescribed to 93% of patients in the usual care group and 90% of patients randomised to the intervention. Overall, the use of cardiovascular medications increased from screening to the end of the study in both groups. From screening to month 6, the percentage of patients prescribed cardiovascular medications increased from 82.4% to 93.3% in the usual care group, and from 81.5% to 89.1% in the intervention group (p < 0.0001 for the increase in medication use from screening in both groups; p = 0.059 between groups). The percentage of patients receiving antihypertensives increased from 80.9% to 91.1%, and 77.9% to 84.7%, among usual care and intervention patients respectively, and the use of serum lipid-reducing agents from 23.9% to 38.8%, and 27.1% to 42.0% respectively (Table 3).

### Adverse events

A total of 34 patients, including two usual care patients who died (causes of death were cardiac arrest and sudden death), experienced 38 serious AEs (SAEs). The most common SAEs were cardiac disorders, including MI (three patients), cardiac arrest (one patient), coronary artery disease (one patient), myocardial ischaemia (one patient) and tachycardia (one patient). AEs reported by ≥ 1% of patients in the usual care or intervention group are listed in Table 4. The percentage of patients who discontinued from the study was 2.8% in the usual care group and 1.9% in the intervention group. The most common reason for discontinuation in both groups was that the patient defaulted (2.0% and 1.4% in the usual care and intervention groups respectively) (Figure 2).

**Table 4:** Adverse events reported by ≥ 1% of patients in the usual care or intervention group

Adverse event *n* (%)	Usual care (*N* = 538)	Intervention (*N* = 565)
Back pain	21 (3.9)	16 (2.8)
Headache	10 (1.9)	12 (2.1)
Cough	6 (1.1)	10 (1.8)
Upper respiratory tract infection	3 (0.6)	8 (1.4)
Osteoarthritis	6 (1.1)	8 (1.4)
Pharyngitis	12 (2.2)	7 (1.2)
Abdominal pain	2 (0.4)	6 (1.1)
Bronchitis	18 (3.3)	6 (1.1)
Dyslipidaemia	8 (1.5)	6 (1.1)

## Discussion

The REACH OUT study demonstrated that a CHD risk evaluation and communication programme mediates a statistically significant reduction in predicted Framingham cardiovascular risk when compared with usual care, among patients with hypertension and moderate cardiovascular risk. At the end of the 6-month study period, patients receiving the intervention had an approximately 10% lower relative predicted 10-year risk of CHD compared with patients given usual care only. Furthermore, patients in the intervention group were observed to have a significantly lower modifiable risk (which excludes the effects of age and gender on predicted CHD risk), relative to those receiving usual care.

### Improvements in risk factor management with intervention vs. usual care

At 6 months, statistically significant differences were observed between the intervention and usual care groups for blood pressure decreases and smoking cessation. Indeed, approximately 8% more patients in the intervention group were reported to have quit smoking during the study than in the usual care group. Reductions in total cholesterol were also numerically greater with the intervention than with usual care, although differences between groups did not reach statistical significance. Framingham risk reductions driven by changes in these factors were impressive in both groups [18% relative reduction with usual care (adjusted absolute reduction 4.9 percentage points); 26% with intervention (adjusted absolute reduction 6.3 percentage points)], particularly given that the majority of patients were already receiving cardiovascular medications at screening. Additionally, improvements in factors such as LDL-C and weight loss, while not reflected in the Framingham risk estimates, nonetheless contribute to a positive change in patients' overall risk profiles. Importantly, approximately twice as many patients in the intervention group achieved blood pressure and LDL-C goals compared with usual care, implying an improvement in the treatment of these risk factors towards guideline-recommended targets by physicians.

While it is not possible to determine precisely the causes of improved CHD risk reduction mediated by the REACH OUT intervention, the observed benefits are likely attributable to behaviour modifications among both physicians and patients. The prescription of cardiovascular medications increased in both the intervention and usual care groups over the study period, which likely contributed to observed risk reductions, although overall increases in the use of cardiovascular medications were slightly lower in the intervention group than in the usual care group. Nonetheless, knowledge of patients' risk status may have prompted physicians to prescribe higher doses of medications or more appropriate therapies. Data for these variables were not collected, however, so firm conclusions cannot be drawn.

Greater interaction with their physician, and increased knowledge and understanding of their risk status, may have motivated patients to adhere to a more healthy diet, increase their exercise, quit smoking or maintain better adherence with medications. The results of the KAB questionnaire demonstrated that patients did indeed experience a small, but statistically significant, increase in their scores for knowledge, attitudes and behaviour, which may have contributed to the observed improvements in CHD risk reduction. Of interest was the finding that patient attitudes appeared to have the greatest correlation with predicted risk, indicating the importance of positively influencing a patient's attitudes to drug therapy and lifestyle changes. In addition, the Heart Health Report was designed to motivate patients by prompting them to consider their future life goals (e.g. their child's wedding, retirement, the birth of a grandchild or travelling the world), which may be a further factor in promoting positive lifestyle changes (38). However, only limited data were collected to evaluate these factors, precluding a full assessment of the causal mechanisms.

Despite the benefits of the intervention on patients' risk factor profiles, the degree of risk reduction in the usual care group was also remarkable. This may be attributed in part to physicians altering their treatment strategies as a result of measuring variables such as blood pressure and lipids during screening procedures, realisation that patients' risk was ≥ 10% (required for inclusion), or because of their awareness of the purpose of the study to evaluate the benefits of overall cardiovascular risk assessment. Furthermore, because of their involvement in a clinical trial, and knowledge that their treatment patterns and achieved risk factor reductions were observed as part of the trial protocol, physicians may have dedicated more time to patient consultations than in their usual clinical practice. These hypotheses are supported by observed increases in the prescription of cardiovascular medications, which were slightly greater in the usual care arm than in the intervention arm. Similarly, patients may have experienced an increase in their motivation to adhere to drug therapy and lifestyle changes because of their involvement in a clinical trial. It is therefore possible that the REACH OUT intervention programme may mediate greater incremental benefits in clinical practice, when compared with true ‘usual care’ outside of a clinical trial setting.

### Modifiable risk assessment

We believe that the assessment of modifiable risk as well as absolute risk is an important clinical concept, which may allow physicians to evaluate more clearly the potential risk reductions that can be achieved with optimal risk factor management. However, it should be noted that estimations of modifiable risk are dependent upon the accepted definition of a ‘normal’ individual. In this study, normal levels of blood pressure and lipids were defined as a systolic blood pressure of 139 mmHg, total cholesterol of 4.9 mmol/l (189 mg/dl) and HDL-C of 1.0 mmol/l (40 mg/dl) for males or 1.2 mmol/l (46 mg/dl) for females (38), which were considered to be levels achievable with the medications readily available in clinical practice. However, clinical outcomes studies have demonstrated benefits of reducing cholesterol to lower levels, particularly among high-risk patients or those with prior CHD (42–44), and observations from subanalyses and epidemiological studies suggest that reducing blood pressure to lower levels may have similar benefits (45–50). In addition, the normal individual for the purposes of this study was defined as not receiving antihypertensive treatment. Thus, in the calculation of modifiable risk, a previously untreated patient would have an increase in their modifiable risk if given antihypertensive therapy during the study, unless this was offset by a considerable reduction in systolic blood pressure. The changes in modifiable risk reported in this study are therefore conservative.

### Implications for clinical practice

Several studies have demonstrated that patient education and support strategies can help to improve patient adherence to medications and mediate improvements in risk factor modification in clinical practice (27–37). These include large trials of nurse-led interventions such as the recent EuroAction trial and the Oxford and collaborators health check (OXCHECK) studies. In EuroAction (27–29), patients with high cardiovascular risk receiving a 16-week, nurse-led cardiovascular prevention and rehabilitation programme experienced significant benefits in terms of risk factor reductions and dietary improvements at one year of follow-up. Overall, there was a comparable absolute difference in blood pressure and LDL-C goal attainment between EuroAction and REACH OUT among high-risk patients receiving the intervention vs. usual care (58% vs. 41%, respectively, for blood pressure; 45% vs. 35%, respectively, for LDL-C). In contrast to REACH OUT, more patients received statins (38% vs. 23%, p = 0.03) and angiotensin-converting enzyme inhibitors (29% vs. 20%, p = 0.02) in the intervention group than in the usual care group, which likely contributed to the benefits observed in EuroAction. In addition, a similar nurse-led, family-oriented cardiovascular screening and lifestyle intervention programme in the earlier British Family Heart Study was associated with a 12% reduction in predicted coronary risk (51). In OXCHECK nurses provided regular patient health checks, which were associated with significantly lower levels of blood pressure (−2.5/1.5 mmHg) and total cholesterol [−0.19 mmol/l (7.3 mg/dl)] vs. the control group at 3 years’ follow-up (30). The authors for the OXCHECK study suggest that these observed reductions in blood pressure would translate to a long-term reduction in risk of MI of 7%, and the decreases in cholesterol to a risk reduction of 6% in men and 13% in women (30).

However, previous studies specifically evaluating the benefits of cardiovascular risk assessment and communication have demonstrated little or no effect on predicted CHD risk (24–26). In the recent Cardiovascular Health Evaluation to improve Compliance and Knowledge among Uninformed Patients (CHECK-UP) study (24), conducted in Canada among patients with previously untreated dyslipidaemia, physicians and patients were informed of the patient's calculated risk through a one-page printout, and patients received ongoing feedback regarding the change in their risk and reductions in ‘cardiovascular age’ (defined as the patient's age minus the difference between their estimated remaining life expectancy and the average remaining life expectancy for Canadian individuals of the same age and sex) as a result of lifestyle changes or drug treatment. The CHECK-UP study demonstrated a statistically significant reduction in 10-year Framingham risk of CVD (for patients without CVD) with the intervention vs. usual care; however, the between-group difference was only approximately −0.6 percentage points (−5.9 with intervention vs. −5.3 with usual care), or about half as effective as the REACH OUT intervention (−1.4 percentage points). Although the REACH OUT study design and patient population are not directly comparable with these earlier studies (24–26), the favourable results observed with the risk evaluation and communication programme may be an indication of the effectiveness of the Heart Health Report for communicating CHD risk, and/or the benefits of the additional follow-up calls from the physician or study nurse.

Elements of the intervention in REACH OUT that focus on increased communication between providers and patients could potentially be provided not only by physicians but also by nursing staff, pharmacists or other healthcare workers, and incorporated relatively easily into routine clinical practice. However, in order to achieve greater improvements in modifiable risk factors than those achieved by patient education/counselling alone (30,34,36,52), it may be beneficial to consider additional interventions as part of a multifactorial approach, for example, adherence interventions (32,35), or CHD risk evaluation as in REACH OUT. In contrast to large-scale interventions such as that in the EuroAction study (27–29) that, if implemented in clinical practice, would require the hiring of additional nursing staff and the associated costs, the REACH OUT intervention could potentially be provided by existing healthcare workers, with only the costs related to the small increase in time devoted to each patient. However, a direct comparison of various investments of clinician's time would be required to fully understand the value of the REACH OUT intervention compared with alternatives. We have no measurement of the exact time that the physician or nurse spent with the patient in REACH OUT (although 78% of patients reported that the physician spent more than 5 min explaining the report), which would be an important factor in determining the cost-effectiveness of the intervention.

Despite the reductions in risk achieved, at the end of the study patients' modifiable risk in both the intervention and usual care groups remained approximately twice as high as for an individual with normal blood pressure and lipids, indicating that additional benefit could be gained from further reductions in modifiable risk factors. Notably, overall levels of blood pressure and LDL-C goal attainment observed in REACH OUT were good when compared with those in usual clinical practice (9,10), but were lower than those achieved in recent clinical trials of antihypertensive and lipid-lowering therapies among patients at varying levels of cardiovascular risk (53–55). In particular, reductions in lipid levels were smaller in REACH OUT than in these other trials, contributing to markedly lower levels of LDL-C goal attainment (53–55). Similarly, the achieved reductions in Framingham risk were smaller than those demonstrated in recent studies evaluating concomitant blood pressure and lipid-lowering therapy (54,56). This may be due to above-average levels of adherence to allocated study medications in these trials, but also suggests that the treatment strategies employed by physicians in REACH OUT and in clinical practice could be improved, perhaps with the use of newer treatments, greater uptitration of medications or the addition of statin therapy in patients with hypertension and additional risk factors (6). Interventions with the potential to improve patient adherence, for instance the use of single-pill combination therapies or blister packs to improve convenience of dosing (32,35,57,58), could provide further benefits to improve risk factor control (59–62).

### Limitations and additional considerations

Concerns were raised by the Ethics Committee in Norway that informing patients of their cardiovascular risk may generate unnecessary anxiety, and the Norwegian committee did not approve the REACH OUT protocol on these grounds. In addition, during the screening process, a number of patients were excluded as they did not wish to be informed of their cardiovascular risk. However, we believe that the REACH OUT study helps to confirm that a realistic perception of cardiovascular risk can motivate patients and physicians to improve risk factor control when combined with a follow-up and support strategy. This concept is supported by a study by Troein et al. (63), which indicated that providing supportive information on cardiovascular risk factors does not increase patient anxiety. Nonetheless, this issue is worthy of consideration and risk communication should be addressed sensitively.

The Framingham calculation was chosen as the method of risk estimation used in REACH OUT as this model is a well understood and effective tool to summarise multiple risk factors and global cardiovascular risk (21,41). It is a limitation of this study, however, that the Framingham model has not been validated broadly in European populations and has been reported to overestimate absolute CHD risk in some studies of European patients (64,65). Furthermore, risk algorithms are not validated for the assessment of treatment effects. Short-term reductions in risk factors leading to reduced estimates of Framingham risk will not necessarily eliminate associated accumulated risks; patients need to maintain these improvements over the long term to reduce their actual risk of disease, and it is uncertain whether the accumulated effects of long-term elevations in risk factors are entirely reversible even with prolonged therapeutic control. In addition, daily biological variations in blood pressure and lipid measurements can affect the precision of risk estimations (66). Risk algorithms can only provide an estimation of long-term risk, and no one method for risk prediction is perfect (67). However, as these limiting factors will be equal in both treatment arms, the between-group differences are still meaningful and we believe that relative changes in predicted risk following intervention can provide a useful indication of the potential benefits if improvements are maintained. In order to accurately determine the true effect of our intervention on cardiovascular outcomes, long-term follow-up studies of patient cardiovascular events are required.

Few women met the REACH OUT study inclusion criterion of 10-year absolute risk ≥ 10%, and those who were included had a high modifiable risk compared with the male population. Thus, the findings from REACH OUT may need to be verified among a greater sample of female patients. Similarly, our findings do not necessarily extend to patients with a history of CHD or diabetes, patients with predicted risk scores < 10% or those who are ≥ 65 years of age, as all of these groups were excluded from the study. An additional study limitation is the relatively short follow-up period of 6 months, which precludes inferences about the long-term impact of the intervention on sustaining improvements in modifiable risk factors and cardiovascular risk reduction.

## Conclusions

The REACH OUT study demonstrates that a patient-focused, physician-implemented CHD risk evaluation and communication programme can effectively lower predicted CHD risk compared with usual care among patients with hypertension and elevated cardiovascular risk, through improvements in modifiable risk factors. This strategy for achieving benefits in risk reduction without dictating drug therapy has wide applicability and is of public health importance. These data support the role of global CHD risk assessment, risk communication and risk factor education for CHD prevention in a multinational, primary care setting. By combining this risk evaluation and communication strategy with more intensive treatment to reduce residual modifiable cardiovascular risk, we believe that substantial improvements in CVD prevention could be achieved in clinical practice.

## Appendix

The REACH OUT study investigators (listed by country).

**Czech Republic:** Dr Tomas Dacik, Dr Marta Fialova, Dr Vaclav Hanka, Dr Radvana Komperova, Dr Marcela Malcova, Dr Vladimir Marek, Dr Jarmila Pavlovicova, Dr Anna Seborova, Dr Anna Uglajova, Dr Milena Hofmanova, Dr Miloslav Fiser, Dr Zdenek Vlasak, Dr Hana Sediva and Dr Helena Hajkova.

**Denmark:** Dr Kim Brockelmann, Dr Niels J. Winther-Pedersen, Dr Preben Kjaerhus, Dr Jesper Isaksen, Dr Jens V. Faaborg-Andersen, Dr Carsten Kjellerup, Dr Mogens Bang, Dr Soren Faurschou and Dr Torben Muller.

**France:** Dr Jacques Marty, Dr Michel Arnould, Dr Leon Grynsztejn, Dr Jean-marie Letzelter, Dr Bernard Perrin, Dr Francois Polard, Dr Pierre Sebastien, Khaled Mekki, Jean Louis Navarre, Dr Claude Burguier, Dr Michel Chay and Dr Christophe Robert.

**Germany:** Dr Lutz Gabriel, Dr Burkhard Wiedeking, Dr Ursula Schax, Dr Gerard Tangerding, Dr Bastian Steinberg, Dr Hans Germann, Dr Christiane Klein, Dr Hannelore Pitule, Dr Volker Aldinger, Dr Klaus Werner, Dr Ottmar Herfert and Dr Thomas Schaefer.

**Greece:** Dr Styliani Argyriadou, Associate Professor Christos Lionis, Dr Antonios Batikas, Dr Marios Chatziarsenis, Dr Athanasios Symeonidis and Dr Dimitris Karanasios.

**The Netherlands:** F. A. A. M. Vermetten, Dr Peter D. M. Coenen, Ivo G. C. M. Bierens, Cees P. Buiks, Aart Leemhuis, W. C. J. van Broekhoven, C. P. Schapers-de Bruin, F. R. de Jong, A. J. M. Boermans, Harry F. C. M. van Mierlo, Willem Veerman and Wilfried A. De Backerm.

**Poland:** Dr Renata Piotrowska, Dr Ireneusz Szymczyk, Dr Andrzej Zajac, Dr Piotr Pietrzak, Dr Adam Ptasinski, Dr Dariusz Horszczaruk, Dr Maria Sledzinska, Dr Irena Gasiorowska, Dr Jacek Kalamarz, Dr Joanna Bocian-Sobkowska, Dr Danuta Widerska-Sobczak, Dr Krzysztof Kliks, Dr Magdalena Kaczmarek and Dr Danuta Kwiatkowska.

**Spain:** Armando Nevado Loro, Jose Luis Martinez Carrasco, Juan Jose Gonzalez Marco, Isabel Saenz del Castillo, Jose Luis Anton Castello, Antonio Rivera Castellano, Amadeo Belles, Juan Jose Martinez Lahuerta, Rafael Alario and Jose Baleriola.

**Sweden:** Dr Goran Hedberg, Dr Sylvia Wide, Dr Roberto M. Zucconi, Dr Margareta Wargelius, Dr Eva Angesjo, Dr Gustav Melin, Bo Sundqvist, Dr Lars Henningsson, Dr Lars Sarhammar, Dr Ann Charlotte Weiderman and Tomas Rahm.

## Supporting information

**Figure S1** Heart Health Report
