# Supplementary material for: A novel programme to evaluate and communicate 10-year risk of CHD reduces predicted risk and improves patients' modifiable risk factor profile
Source: Int J Clin Pract. 2008 Oct;62(10):1484–98. doi: 10.1111/j.1742-1241.2008.01872.x (PMC2658032; doi:10.1111/j.1742-1241.2008.01872.x)
Supplement: Supplementary file 1 [file ijcp0062-1484-SD1.doc]

**Supplementary material (to appear online only)**

**Figure I:** Heart Health Report

Reprinted from *Contemp Clin Trials*, Vol 28, Benner JS, Cherry SB, Erhardt L et al., Rationale, design, and methods for the risk evaluation and communication health outcomes and utilization trial (REACH OUT), Pages 662-73, Copyright (2007), with permission from Elsevier (http://www.sciencedirect.com/science/journal/15517144)
